# Analysis of preterm deliveries below 35 weeks' gestation in a tertiary referral hospital in the UK. A case-control survey

**DOI:** 10.1186/1756-0500-3-119

**Published:** 2010-04-28

**Authors:** Wei Yuan, Anne M Duffner, Lina Chen, Linda P Hunt, Susan M Sellers, Andrés López Bernal

**Affiliations:** 1 University of Bristol, Department of Clinical Science at South Bristol (Obstetrics and Gynaecology), St Michael's Hospital, Southwell St. Bristol, BS2 8EG. UK; 2 University of Bristol, Department of Social Medicine, Canynge Hall, Whiteladies Road, Bristol, BS8 2PR. UK; 3 University of Bristol, Dept of Clinical Sciences at South Bristol, Institute of Child Life and Health, Education Centre, Upper Maudlin Street, Bristol, BS2 8AE. UK; 4 University Hospitals Bristol, Obstetrics and Gynaecology, St Michael's Hospital, Southwell St. Bristol, BS2 8EG. UK

## Abstract

**Background:**

Preterm birth remains a major public health problem and its incidence worldwide is increasing. Epidemiological risk factors have been investigated in the past, but there is a need for a better understanding of the causes of preterm birth in well defined obstetric populations in tertiary referral centres; it is important to repeat surveillance and identify possible changes in clinical and socioeconomic factors associated with preterm delivery. The aim of this study was to identify current risk factors associated with preterm delivery and highlight areas for further research.

**Findings:**

We studied women with singleton deliveries at St Michael's Hospital, Bristol during 2002 and 2003. 274 deliveries between 23-35 weeks' gestation (preterm group), were compared to 559 randomly selected control deliveries at term (37-42 weeks) using standard statistical procedures. Both groups were >80% Caucasian. Previous preterm deliveries, high maternal age (> 39 years), socioeconomic problems, smoking during pregnancy, hypertension, psychiatric disorders and uterine abnormalities were significantly associated with preterm deliveries. Both lean and obese mothers were more common in the preterm group. Women with depression/psychiatric disease were significantly more likely to have social problems, to have smoked during pregnancy and to have had previous preterm deliveries; when adjustments for these three factors were made the relationship between psychiatric disease and pregnancy outcome was no longer significant. 53% of preterm deliveries were spontaneous, and were strongly associated with episodes of threatened preterm labour. Medically indicated preterm deliveries were associated with hypertension and fetal growth restriction. Preterm premature rupture of the membranes, vaginal bleeding, anaemia and oligohydramnios were significantly increased in both spontaneous and indicated preterm deliveries compared to term controls.

**Conclusions:**

More than 50% of preterm births are potentially preventable, but remain associated with risk factors such as increased uterine contractility, preterm premature rupture of the membranes and uterine bleeding whose aetiology is unknown. Despite remarkable advances in perinatal care, preterm birth continues to cause neonatal deaths and long-term morbidity. Significant breakthroughs in the management of preterm birth are likely to come from research into the mechanisms of human parturition and the pathophysiology of preterm labour using multidisciplinary clinical and laboratory approaches.

## Background

Preterm birth remains a major cause of perinatal mortality and morbidity and efforts to predict and prevent its occurrence are difficult because of our lack of understanding of the biochemical mechanism of labour and the multiplicity of medical and socioeconomic factors associated with preterm delivery [1]. The incidence of preterm birth (deliveries before 37 weeks' gestation) ranges from 6-8% in Europe, Australia and Canada [2,3], to 9-12% in Asia, Africa and the United States [4,5]. Preterm birth is recognised as a worldwide problem responsible for more than 80% of neonatal deaths and more than 50% of long term morbidity in the surviving infants [6,7]. The incidence of preterm birth has remained relatively constant over the past three decades and there are worrying trends that it is on the increase [5,8]. It is important to know whether risk factors have changed over the years and to look for new clinical and socioeconomic risks. This information is necessary to guide further research in this area. Epidemiological studies have identified a clear association between preterm birth and previous preterm delivery [9], preterm premature rupture of the membranes [10] and maternal smoking during pregnancy [11]. However the association between preterm birth and other maternal and fetal complications of pregnancy is less consistent, due to social, ethnic and demographic differences amongst the populations studied [7]. In this study we have carried out a detailed comparison of preterm and term deliveries in a relatively homogeneous obstetric population attending a tertiary referral maternity hospital, to highlight areas where further research or intervention is needed in order to prevent preterm birth and improve perinatal outcome.

## Methods

### Study population

This survey was done at St Michael's Hospital, Bristol. This is a tertiary referral maternity centre with approximately 5500 deliveries a year; preterm deliveries (under 37 weeks' gestation) accounted for approximately 8% of all births during 2002 and 2003. In this survey we have focused on deliveries between 23-35 weeks' gestation (3% of all births) because these account for most of the perinatal mortality and morbidity and so improvements in management are likely to have a strong impact on neonatal outcome. We obtained lists of all deliveries below 35 weeks gestation from the hospital database for two complete years 2002 and 2003 (n = 274). We used computer generated random numbers to draw separate samples from lists of all term babies delivered within each of the 2 years, using term: preterm ratios of 2:1 to increase power; 559 out of 8204 term babies were included. Gestational age was based either on certain dates or a dating scan. Antenatal care was undertaken by an obstetrician or community midwife or a combination of the two professionals and followed standard UK practice.

This study was approved by the Central & South Bristol Research Ethics Committee.

### Statistical analysis

Initial maternal and newborn information was taken from the hospital computer databases (STORK and Vermont Oxford Network). The hospital medical records of each delivery were retrieved and analysed in detail by a trained research midwife (AMD) using a structured proforma. Information recorded was anonymised by assigning a unique project number to each delivery. Data on maternal and fetal characteristics at birth were recorded, including maternal age and gestational age at delivery, ethnicity, gestational age at booking, complications of pregnancy and delivery, birth weight, Apgar score and baby gender. A wide range of potential risk factors - smoking and drinking status before and during pregnancy, history of drug use, socioeconomic status, past obstetric history (including previous history of termination, miscarriage and preterm birth) were recorded. Maternal body mass index (BMI) at booking was calculated using weight (kg)/height (m)^2^. The definition of lean was BMI <20 kg/m^2 ^and obese BMI ≥ 30 kg/m^2^. Data was entered into a Microsoft excel 2003 database and imported into STATA version 10 for analysis.

Comparisons between cases and controls were made using standard statistical procedures. Continuous variables were summarised by mean (SD) except for gestational age, which was not normally distributed, where the median (range) was used instead. Means were compared using unpaired Student's t-tests. For categorical variables Chi-squared tests were used and two-tailed Fisher's Exact tests for 2 × 2 tables where expected frequencies were small. A 5% level of significance was used throughout. Multiple logistic regression was used to adjust for confounding factors and compute odd-ratios.

## Results

The control and preterm groups had very similar ethnicity and maternal age but, as expected, multiple births were more common in the preterm group (Table 1). Eight of the multiple pregnancies were the result of assisted reproduction treatment (IVF). Since the obstetric management of multiple pregnancies differs from that of singleton pregnancies, we excluded all the multiple pregnancies from further analysis.

**Table 1:** General characteristics of the study groups.

	Control (≥ 37 weeks' gestation)	Preterm (<35 weeks' gestation)	P value
Number of deliveries	559	274	

Gestational age at delivery (completed weeks)	Median 40 (Range 37 - 42)	Median 33 (Range 23 - 34)	Not Applicable

*Ethnicity*			

Caucasian	464 (83.0%)	229 (83.6%)	0.46
	
Asian	45 (8.1%)	17 (6.2%)	
	
African	33 (5.9%)	22 (8.0%)	
	
Others	17 (3.0%)	6 (2.2%)	

Mean maternal age (years)	28.8 (SD 6.04) (Range 15-44)	29.7 (SD 6.46) (Range16-46)	0.045

*Distribution of births*			

Singletons	553 (98.9%)	228 (83.2%)	<0.001
	
Twins	6 (1.1%)	44 (16.1%)	
	
Triplets	0	2 (0.7%)	

### Maternal age and BMI

Previous reports have indicated that extremes of maternal age and low maternal weight predispose to preterm birth and we have confirmed that the incidence of preterm birth in women over 39 years of age remains significantly higher than in younger women (Table 2); however the proportion of teenage mothers in the two groups was similar. Both lean and obese mothers were more common in the preterm group (odds ratios 1.75 (95%CI 1.02 to 3.01) and 1.41 (95% 0.92-2.15) respectively; reference category was the normal BMI group), but the overall difference did not reach statistical significance (Table 2).

**Table 2:** Demographic and socioeconomic parameters in singleton pregnancies.

	Control (≥ 37 weeks' gestation)	Preterm (<35 weeks' gestation)	P value
Number of deliveries	553	228	

Median gestational age at delivery (completed weeks) and range	40 (Range 37-42)	33 (Range 23 - 34)	Not Applicable

*Maternal age*			

Mean maternal age at delivery (years)	28.8 (SD 6.04) (Range 15-44)	29.4 (SD 6.69) (Range16-46)	0.24

≤ 18 years	33 (6.0%)	13 (5.7%)	
	
19-39 years	509 (92.0%)	199 (87.3)	0.002
	
>39 years	11 (2.0%)	16 (7.0%)	(Comparison of proportions of mothers >39 years P < 0.001)

*Body mass index*			

Mean BMI at booking (kg/m^2^)	25.03 (SD 5.29) (Range 16-49) (n = 531)	25.8 (SD 6.71) (Range 15-60) (n = 195)	0.10

Lean <20	42 (7.9%)	24 (12.3%)	0.054
	
Normal 20 - 29.9	402 (75.7%)	131 (67.2%)	
	
Obese ≥30	87 (16.4%)	40 (20.5%)	

*Socioeconomic*			

With social problems	60/548 (10.9%)	50/218 (22.9%)	<0.001

Smoking during pregnancy	114/553 (20.6%)	83/226 (36.7%)	<0.001

Drinking Alcohol during pregnancy	180/550 (32.7%)	75/228 (32.9%)	0.96

Using drugs during pregnancy	20/553 (3.6%)	12/226 (5.3%)	0.28

### Socioeconomic factors

The proportion of women with at least one social problem (low income; poor housing; unsupported or single parent) was significantly higher in the preterm than in the control group (Table 2). Smoking before conception was not associated with preterm birth (data not shown); however the proportion of women who smoked during pregnancy was significantly higher in the preterm than in the control group. The proportion of smokers in women with and without social problems was 58.2% and 19.6%, respectively. The proportions of women who consumed alcohol or admitted using any recreational drugs (cannabis, amphetamines, barbiturate, crack, cocaine, heroin, methadone, ecstasy) during pregnancy were similar in the control and preterm groups.

### History of previous pregnancies

About 70% of women in both groups had been pregnant previously and more than 56% had had previous deliveries (Table 3). The proportion of women with a previous preterm delivery (between 20 and 37 weeks' gestation) in the preterm group was significantly higher than in the control group. Analysis of previous pregnancies under 20 weeks' gestation revealed that terminations of pregnancy were significantly more common in the preterm group; however the proportion of spontaneous miscarriages in the two groups was similar.

**Table 3:** Previous pregnancies.

	Control (≥37 weeks' gestation)	Preterm (<35 weeks' gestation)	P value
Number of deliveries	553	228	

Gravida 0	176 (31.8%)	62 (27.2%)	0.20
Gravida >0	377 (68.2%)	166 (72.8%)	

Para 0	239 (43.2%)	100 (43.9%)	0.87
	
Para >0	314 (56.8%)	128 (56.1%)	

Number of women with previous preterm delivery (≥ 20 and <37 weeks' gestation).	30 (5.4%)	58 (25.4%)	<0.001

Termination of pregnancy (< 20 weeks' gestation)	88 (15.9%)	55 (24.1%)	0.007

Spontaneous miscarriage (< 20 weeks' gestation)	30 (5.4%)	9 (3.9%)	0.39

### Pre-existing medical conditions

The most common pre-existing medical conditions in the control and preterm deliveries are listed in Table 4. Hypertension (blood pressure >140/90 mmHg), psychiatric disorders and uterine abnormalities were significantly associated with preterm delivery. A history of cervical incompetence was found only in the preterm group. Multivariable analysis revealed that women with psychiatric disorders were significantly more likely to have social problems (P < 0.001), to have smoked during pregnancy (P < 0.001) and to have had previous preterm deliveries (P = 0.007). When adjustments were made for the effect of these three factors the relationship between psychiatric disorders and preterm birth was no longer significant (odds-ratio [95%CI] reduced from 1.6 [1.1-2.4] to 1.2 [0.8-1.8])

**Table 4:** Pre-existing medical conditions.

	Control (≥37 weeks' gestation)		Preterm (<35 weeks' gestation)		P value
Number of deliveries	553		228		

Anaemia	103 (18.6%)		45 (19.7%)		0.72

Respiratory problems	89 (16.1%)		39 (17.1%)		0.73

Depression/Psychiatric Disease	83 (15.0%)		50 (21.9%)		0.019

Hypertension	51 (9.2%)		34 (14.9%)		0.020

Renal Disease	34 (6.1%)		18 (7.9%)		0.37

Sexually transmitted diseases	33 (6.0%)		16 (7.0%)		0.58

Cardiac disease	24 (4.3%)		9 (3.9%)		0.80

Thyroid Disease	14 (2.5%)		3 (1.3%)		0.29

IVF	12 (2.2%)		6 (2.6%)		0.70

Diabetes	11 (2.0%)		3 (1.3%)		0.52

Hepatitis B	10 (1.8%)		5 (2.2%)		0.72

Bleeding disorders	8 (1.4%)		3 (1.3%)		>0.99*

Uterine abnormalities	10 (1.8%)		14 (6.1%)		0.001

Cervical incompetence/cervical suture	0		5 (2.2%)		0.002*

*Two-tailed Fisher's Exact test

### Complications of pregnancy

The incidence of maternal, fetal and other complications of pregnancy is shown in Table 5. Episodes of threatened preterm labour were the most common maternal complication in preterm deliveries, and occurred almost exclusively in the preterm group. Vaginal bleeding, anaemia (haemoglobin < 10.5 g/dl) and proteinuria (1+ or more on dipstick, excluding urinary tract infection) occurred in both control and preterm pregnancies, but were significantly increased in the latter. Hypertension with proteinuria (pre-eclampsia) was significantly increased in the preterm group. Hypertension without proteinuria (pregnancy induced hypertension) was low in both groups. Raised alpha feto protein (AFP) levels were more common in preterm pregnancies, but the incidence was low. Other common complications such as urinary tract infection (UTI), the presence of pathogens in high vaginal swabs (HVS) and the observation of reduced fetal movements were similar in both groups (Table 5).

**Table 5:** Complications of pregnancy.

	Control (≥37 weeks' gestation)	Preterm (<35 weeks' gestation)	P value
Number of deliveries	553	228	

*Maternal complications*			

Threatened preterm labour	17/553 (3.1%)	126/228 (55.3%)	<0.001

Vaginal bleeding	130/553 (23.5%)	117/228 (51.3%)	<0.001

Anaemia	53/513 (10.3%)	76/224 (33.9%)	<0.001

Hypertension with proteinuria	43/514 (8.4%)	52/224 (23.2%)	<0.001

Hypertension without proteinuria	13/514 (2.5%)	4/224 (1.8%)	0.54

Urinary tract infection	56/514 (10.9%)	35/224 (15.6%)	0.072

HVS B Strep.	26/514 (5.1%)	12/224 (5.4%)	0.87

HVS Other organisms	70/514 (13.6%)	35/224 (15.6%)	0.47

Hyperemesis	16/514 (3.1%)	3/224 (1.3%)	0.16

Gestational Diabetes	14/514 (2.7%)	4/224 (1.8%)	0.45

Late booker	16/513 (3.1%)	8/223 (3.6%)	0.74

High Down's Risk	16/513 (3.1%)	7/224 (3.1%)	>0.99

Raised AFP	4/513 (0.8%)	6/224 (2.7%)	0.075*

*Fetal complications*			

Reduced fetal movements	140/514 (27.2%)	76/224 (33. 9%)	0.066

Oligohydramnios	49/514 (9.5%)	65/224 (29.0%)	<0.001

Fetal growth restriction	12/514 (2.3%)	41/224 (18.3%)	<0.001

Large for dates	27/514 (5.3%)	8/224 (3.6%)	0.32

Fetal abnormalities	14/553 (2.5%)	15/228 (6.6%)	0.007

*Other complications*			

Preterm premature rupture of membranes	5/553 (0.9%)	100/228 (43.9%)	<0.001

Prolonged rupture of membranes	49/553 (8.9%)	49/228 (21.5%)	<0.001

*Two-tailed Fisher's Exact test

Fetal complications were more frequent in the preterm group, especially oligohydramnios, followed by fetal growth restriction and fetal abnormalities (including both structural and chromosomal abnormalities) (Table 5). Preterm premature rupture of the membranes (PPROM) and prolonged rupture (> 48 hours) of the membranes were significantly more common in the preterm group.

Multivariable analysis indicated that women with pre-eclampsia were more likely to have fetal growth restriction; moreover each of the two factors was an effect modifier for the other (supported by significant interaction in logistic regression P = 0.026). For example, the association of fetal growth restriction with preterm birth was much greater in the presence of pre-eclampsia than in its absence (OR 45.4 [95% CI 5.8-354.6] versus 3.71 [1.65-8.33], respectively). Women with both premature and prolonged rupture of the membranes were the most likely to have oligohydramnios (P < 0.001). Finally, women with vaginal bleeding were more likely to have PPROM or episodes of threatened preterm labour (P < 0.001 for both).

### Labour and delivery

**Figure 1:**
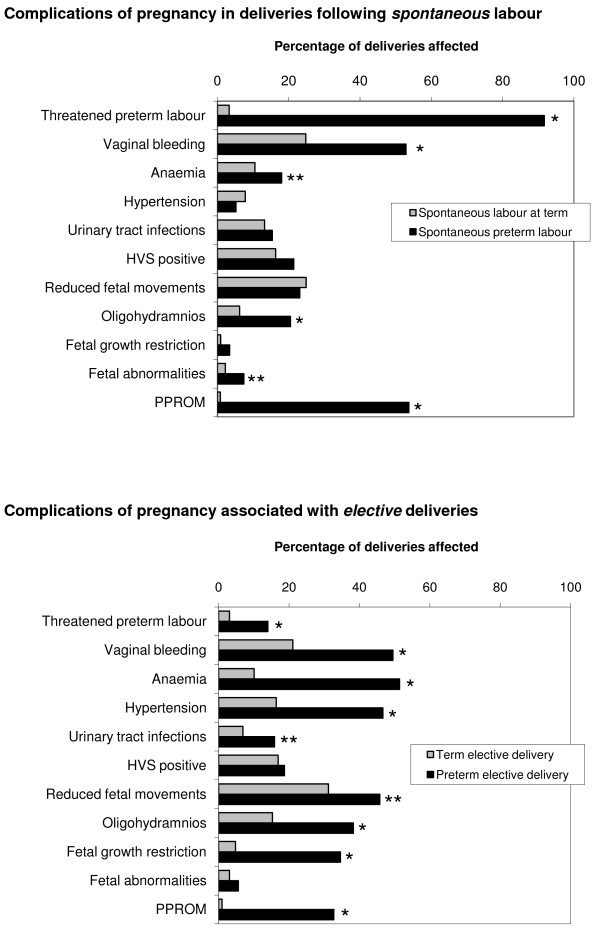
**Complications of pregnancy in spontaneous and medically indicated elective preterm deliveries (23-35 weeks' gestation) compared with spontaneous and elective term controls (≥37 weeks' gestation)**. HVS: high vaginal swab. PPROM: preterm premature rupture of the membranes. Significant differences: * p < 0.001; ** p < 0.05.

65% of women in the control group went into spontaneous labour and 35% had an elective delivery (caesarean section or induction of labour) indicated for medical or obstetric reasons. In the preterm group 53% of women had spontaneous labour and 47% were delivered electively (P = 0.002). When the incidence of complications of pregnancy is presented separately in spontaneous and elective deliveries (Figure 1) very different patterns can be observed. Spontaneous preterm deliveries were clearly associated with threatened preterm labour, PPROM and vaginal bleeding. The occurrence of one or more episodes of vaginal bleeding at any stage of pregnancy was relatively common in both term and preterm spontaneous deliveries, but the incidence was significantly higher in the preterm group. By contrast, elective preterm deliveries were strongly associated with anaemia, hypertension and fetal growth restriction (Figure 1). Interestingly, vaginal bleeding, oligohydramnios, PPROM and, to a lesser extent, threatened preterm labour were significantly increased in both spontaneous and elective preterm deliveries.

### Neonatal outcome

Metabolic complications including jaundice, hypoglycaemia and hypothermia were relatively common in both control and preterm groups, although the incidence was significantly higher in the latter (data not shown). Severe complications of prematurity such as respiratory distress (56%), patent ductus arteriosus (11%), intracerebral damage (9%) and necrotising enterocolitis (4.5%) were diagnosed almost exclusively in the preterm group. There were 15 stillbirths, 21 neonatal deaths (deaths within 28 days of birth) and 6 postneonatal deaths (after 28 days) in the preterm group compared to only 2 stillbirths, 2 neonatal deaths and 1 postneonatal death in the control group. The main causes of death in the preterm group were severe prematurity, infection and congenital abnormalities; in the control group deaths were associated with congenital abnormalities.

## Discussion

This manuscript provides information on clinical factors associated with preterm delivery in a tertiary referral hospital in the UK. The study benefits from its relatively homogeneous ethnic population and its setting in a maternity hospital with unified management guidelines in the central delivery suite. Moreover, it highlights the main risk factors that remain associated with preterm birth, and emphasizes the need to promote research into the basic mechanisms of parturition as the best way to develop effective management for spontaneous preterm labour.

In our predominantly Caucasian population, teenage pregnancies were not significantly associated with preterm birth; however, our data confirm that older mothers have an increased risk of preterm delivery [12,13]. A low BMI is associated with increased risk of spontaneous preterm birth [14] and this was reflected in our population. Previous preterm delivery is a strong risk factor for subsequent preterm birth, with a five fold higher rate of previous preterm delivery in the preterm compared to the control group; this indicates that maternal factors are important, however the mechanism remains unclear. A history of preterm delivery has long been recognised as a strong risk factor for subsequent preterm birth [9] and is the basis for most risk scoring systems [15]. Moreover, our data confirm that hypertension and fetal growth restriction are major predisposing factors for elective preterm delivery [7,16].

Episodes of threatened preterm labour were strongly associated with spontaneous preterm deliveries. Thus, spontaneous preterm labour is characterised by remarkable uterine hyperactivity. The mechanism requires further investigation but it probably results from increased sensitivity of the uterus to stimulatory agonists such as oxytocin, prostaglandins or other endogenous mediators [1], as well as a premature loss of inhibitory pathways involving myometrial ion channels [17]. We have recently shown that spontaneous preterm labour is associated with increased GTP bound RHO proteins in myometrial tissue, a pathway for enhanced uterine contractility through 'calcium sensitization'[18]. Uterine contractions are the most common presenting sign of preterm labour but in a high percentage of women the contractions stop without the need for tocolytic treatment. Separating imminent spontaneous preterm labour from recurrent but transient episodes of uterine contractions remains a major clinical challenge.

Bleeding in pregnancy was strongly associated with both spontaneous and elective preterm deliveries. Women with vaginal bleeding have an increased risk of induction of labour and caesarean section and the condition is associated with other pregnancy complications such as PPROM, oligohydramnios and fetal growth restriction [19]. The mechanism by which intrauterine bleeding may lead to spontaneous preterm labour is not known, but it has been proposed that thrombin activation in the decidua leads to uterine contractions [20,21]. Moreover thrombin increases matrix metalloproteinase activity in the fetal membranes providing a link between intrauterine bleeding and rupture of the membranes [21,22].

Anaemia is one of the most common nutritional problems in pregnant women throughout the world and, after smoking, is the most important preventable risk factor for preterm birth. Our data show that even moderate anaemia (< 10.5 g/dl) in pregnancy is associated with preterm birth and this agrees with observations in other tertiary referral hospitals [23]. Ascending intrauterine infection is often quoted as a pathogenic mechanism for preterm labour [7], however in our survey the proportion of women with bacterial pathogens in high vaginal swabs was similar in the control and preterm groups.

Preterm babies continue to die in the perinatal period or have severe neonatal complications which predispose to a high incidence of neurodevelopmental impairments and sensory deficits. The early administration of glucocorticoids to the mother and impressive advances in neonatal care have steadily improved neonatal survival rates over the past three decades; however it is unrealistic to expect that improvements in neonatal intensive care will decrease neonatal mortality and the sequelae of prematurity much further [24]. The onus is now on understanding the causes and mechanisms of parturition, so that spontaneous preterm labour can be prevented and preterm birth is only allowed to happen electively for the benefit of the mother and her baby [25]. The proportion of elective preterm deliveries in our survey is considerably higher than in similar UK and European hospital populations in the 1970s and 1980s [6,26]. This reflects a growing confidence among young obstetricians that justified intervention in preterm pregnancies results in good obstetric and neonatal outcome, but significant morbidity should not be forgotten.

This study has limitations because it has surveyed a population of several hundred women in a single tertiary hospital. The factors associated with preterm birth would be better addressed through prospective study of a very large geographical cohort. Furthermore, we believe that over the next decade epidemiological data will be supplemented by advances in uterine physiology and materno-fetal endocrinology which will improve our understanding of human parturition and help devise successful strategies to prevent preterm labour.

## Conclusions

The data from this study show that more than 50% of preterm births follow spontaneous preterm labour and further research to clarify the mechanism by which risk factors such as increased uterine contractility, premature rupture of the membranes and uterine bleeding result in preterm labour will be clearly beneficial. Moreover, it is important to address the major causes of elective preterm delivery, namely hypertensive disorders and intrauterine growth restriction. This may be achieved through the discovery of the aetiology of pre-eclampsia and a better understanding of the control of fetal growth and placental function. The reduction of spontaneous preterm labour is a realistic aim; however our lack of knowledge of the process of labour is a major handicap in devising effective strategies. It is essential to promote research into the physiological and physiopathological pathways that increase uterine activity during pregnancy. The combination of laboratory and clinical research will provide the necessary breakthroughs to improve the prevention of preterm birth.

## Competing interests

The authors declare that they have no competing interests.

## Authors' contributions

WY: Contributed to preparation and analysis of the data. AMD: Substantial contribution to acquisition of data. LC: Substantial contribution to analysis of the data. LPH: Contributed to design of the study and statistical analysis of the data. SMS: Contributed to clinical interpretation of the data and writing of the manuscript. ALB: Design of the study and writing of the manuscript.

All authors have read and approved the final manuscript.

## Authors' information

WY: MSc. PhD student, Department of Clinical Science at South Bristol (Obstetrics and Gynaecology). University of Bristol. AMD: Registered Midwife, Department of Obstetrics and Gynaecology, St Michael's Hospital, Bristol. LC: MSc. Research associate, Department of Social Medicine, University of Bristol. LPH: PhD, CStat, Senior Lecturer in Medical Statistics, Dept of Clinical Sciences at South Bristol. University of Bristol. SMS, MD, FRCOG, Consultant Obstetrician, University Hospitals Bristol, Obstetrics and Gynaecology, St Michael's Hospital. ALB, MD, D Phil, Professor of Human Reproductive Biology, Department of Clinical Science at South Bristol (Obstetrics and Gynaecology). University of Bristol.
